# High-throughput droplet microfluidics screening and genome sequencing analysis for improved amylase-producing *Aspergillus oryzae*

**DOI:** 10.1186/s13068-023-02437-6

**Published:** 2023-11-29

**Authors:** Qinghua Li, Jinchang Lu, Jingya Liu, Jianghua Li, Guoqiang Zhang, Guocheng Du, Jian Chen

**Affiliations:** 1 Science Center for Future Foods, Jiangnan University, 1800 Lihu Road, Wuxi, 214122 Jiangsu China; 2 National Engineering Research Center for Cereal Fermentation and Food Biomanufacturing, Jiangnan University, 1800 Lihu Road, Wuxi, 214122 Jiangsu China; 3 School of Biotechnology and Key Laboratory of Industrial Biotechnology, Ministry of Education, Jiangnan University, 1800 Lihu Road, Wuxi, 214122 Jiangsu China; 4 The Key Laboratory of Carbohydrate Chemistry and Biotechnology, Ministry of Education, Jiangnan University, 1800 Lihu Road, Wuxi, 214122 Jiangsu China

**Keywords:** *Aspergillus oryzae*, High-throughput screening, Microfluidics, Flow cytometry, Genome resequencing, α-Amylase

## Abstract

**Background:**

The exceptional protein secretion capacity, intricate post-translational modification processes, and inherent safety features of *A. oryzae* make it a promising expression system. However, heterologous protein expression levels of existing *A. oryzae* species cannot meet the requirement for industrial-scale production. Therefore, establishing an efficient screening technology is significant for the development of the *A. oryzae* expression system.

**Results:**

In this work, a high-throughput screening method suitable for *A. oryzae* has been established by combining the microfluidic system and flow cytometry. Its screening efficiency can reach 350 droplets per minute. The diameter of the microdroplet was enlarged to 290 µm to adapt to the polar growth of *A. oryzae* hyphae. Through enrichment and screening from approximately 450,000 droplets within 2 weeks, a high-producing strain with α-amylase increased by 6.6 times was successfully obtained. Furthermore, 29 mutated genes were identified by genome resequencing of high-yield strains, with 15 genes subjected to editing and validation. Two genes may individually influence α-amylase expression in *A. oryzae* by affecting membrane-associated multicellular processes and regulating the transcription of related genes.

**Conclusions:**

The developed high-throughput screening strategy provides a reference for other filamentous fungi and *Streptomyces*. Besides, the strains with different excellent characteristics obtained by efficient screening can also provide materials for the analysis of genetic and regulatory mechanisms in the *A. oryzae* expression system.

**Supplementary Information:**

The online version contains supplementary material available at 10.1186/s13068-023-02437-6.

## Introduction

*Aspergillus oryzae* is a substantial strain in the fermentation industry and plays a vital role in food, medicine, agriculture, and feed [1]. In recent years, *A. oryzae* has shown great potential and competitive advantages to be developed into an excellent expression system due to its extensive substrate utilization, robust protein synthesis and secretion capacity, and substantial post-translational modification [2]. In addition, *A. oryzae* is generally recognized as safe (GRAS) by the US Food and Drug Administration (FDA), meeting the safety needs of industrial production [3]. Therefore, researchers have invested much work in constructing and developing high-efficiency *A. oryzae* expression systems and achieved progress by establishing genetic transformation systems and genome editing technologies [4, 5]. The rapid development of omics research including genomics and transcriptomics also accelerated the exploration of potentially relevant mechanisms [6–8]. The *A. oryzae* expression system has played an essential role in expressing proteins, organic acids, and secondary metabolites, but further development, optimization, and popularization still face challenges [9]. Wild strains are rarely directly used for industrial-scale production due to their low yield and poor tolerance to harsh industrial conditions. In addition, the regulatory mechanism for the huge difference in the expression levels of homologous and heterologous proteins is complex and unclear [10]. The mutagenesis screening and the optimization of industrial-scale fermentation conditions can, to some extent, promote the industrial application of *Aspergillus* [11–13]. However, labor-intensive screening methods based on colony plate and shaking culture are inefficient, impeding the rapid acquisition of superior strains [13, 14]. Therefore, it is crucial to establish a high-efficiency screening technology suitable for *A. oryzae*.

Currently, high-throughput screening technology is becoming more mature and widely used to screen microorganisms efficiently [15]. Flow Cytometry (FC) can quickly analyze multiple parameters of a single cell and classify target cells in various ways, which is a promising high-throughput screening technology [16, 17]. However, FC can only screen *Aspergillus* spores in the early germination stage, but not for filamentous forms of *Aspergillus* [18]. Moreover, FC is used chiefly to analyze intracellular or membrane-bound products that generate fluorescent signals associated with target compounds, while it is challenging to analyze extracellular products [19]. Thomas Beneyton et al. used microdroplets to achieve high-throughput screening of secreted protein based on fluorescence intensity detection, laying the foundation for high-throughput screening of *Aspergillus* [14, 20]. Therefore, it is logical to establish a high-throughput screening method based on a microfluidic system, as it overcomes the limitations of traditional FC [21, 22]. In a microfluidic system, the microdroplet is a micro-culture chamber that provides mycelium growth and product accumulation until the end of the screening [23]. Hence, the stabilization of microdroplets is one of the indispensable conditions in the high-throughput screening process [24]. However, the polar growth of *A. oryzae* mycelium pierces the droplets, limiting the time for product accumulation and droplet detection and sorting, and seriously affecting the screening method's accuracy and application range [14]. To avoid microdroplet puncture, Zhou et al. designed a biocompatible core–shell droplet-based microfluidic (REPID) system, which has good stability for spore culture as well as protein synthesis and secretion [25]. However, the low dissolved oxygen is a problem that cannot be ignored, and the addition of the shell layer may further affect the dissolved oxygen in the droplets, thereby affecting the normal growth and metabolic network of mycelia.

In this study, to meet the culture time of hyphae in the microdroplets, the diameter of the microdroplet was enlarged to 290 µm and applied in high-throughput screening of α-amylase producing *A. oryzae*. Furthermore, the screening efficiency and subsequent purification and separation operations were further improved by combining with FC. The screening period was shortened from 1 month to 2 weeks. It provided a reference value for high-throughput screening of polar growing filamentous fungi. Besides, genome re-sequencing and genetic validation were conducted to explore the potential mechanisms to guide the construction of high-producing strains.

## Results and discussion

### Large-sized microdroplet generation and process optimization

The polar growth of mycelium is a typical feature of *A. oryzae*, and it is also a problem that cannot be ignored in high-throughput screening [26]. Rapidly growing hyphae may puncture the droplets and fail the screening before the droplets accumulate enough target product. To achieve high-throughput screening of *A. oryzae*, larger microdroplets generated using a droplet generation chip fabricated with a depth of 250 µm were used to provide growth time and accumulation targets product [14]. The type of oil phase was first optimized to generate microdroplets stably and uniformly. FC40 was too viscous to generate microdroplets with larger diameters in the chip; while Novec7500 and Bio-Rad droplet-forming oils could both generate microdroplets with a diameter of 200–250 μm (Additional file 1: Fig. S1). Nonetheless, the microdroplets generated using the Bio-Rad method exhibited partial fusion and rupture within a short timeframe (Additional file 1: Fig. S1a). Therefore, Novec7500 was selected for microdroplet generation in subsequent experiments as the microdroplets generated with Novec7500 were uniform and stable (Additional file 1: Fig. S1b).

**Fig. 1:**
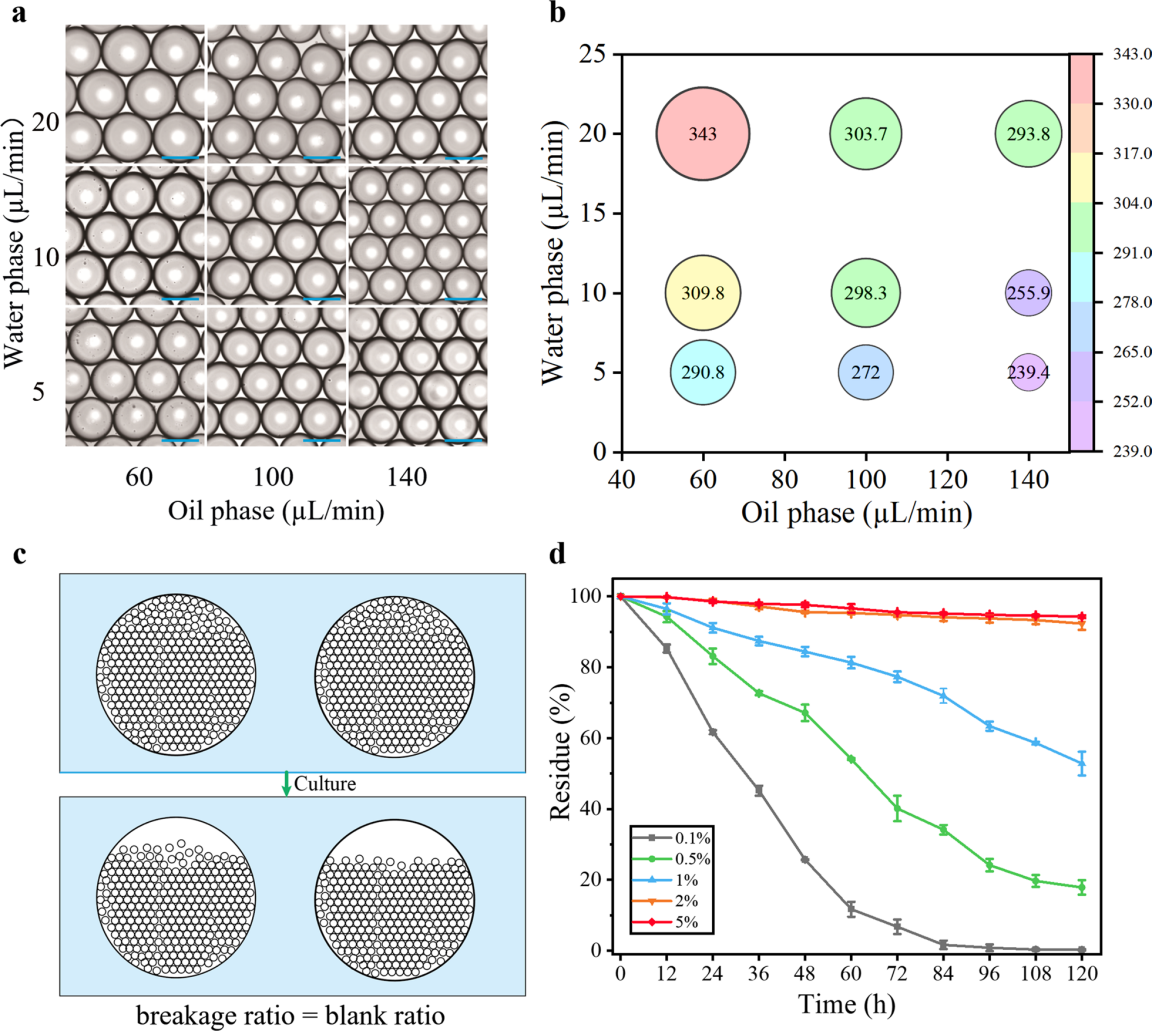
Optimization of conditions for microdroplet generation. **a** Optimization of microdroplet diameters at different flow rate ratios of the oil-to-water phase. Scale bar: 250 μm. **b** Relationship between microdroplet diameters and flow rate of oil-to-water phases. The number in the circle indicates the droplet diameter, which was calculated from microscopy images using n = 100 droplets. Unit: μm. **c** Schematic diagram for calculation of droplet breakage rate; **d** effect of surfactant on droplet stability

Besides, the flow rates of the droplet-generating oil and water phases were optimized to obtain microdroplets with larger sizes. The microdroplet diameter is proportional to the water velocity and inversely proportional to the oil velocity, and the diameter of the generated microdroplets ranges from 239.4 µm to 343 µm (Fig. 1a, b). The corresponding speeds of microdroplet generation varied from 50 droplets per second to 200 droplets per second. In general, microdroplet stability tends to decrease as the diameter of the microdroplets increases. The larger size of microdroplets results in greater inertia, necessitating higher voltages for efficient sorting. However, increasing the sorting voltage may lead to a reduced rate of spore regeneration after sorting. Considering the stability of large droplets and the regeneration rate of spores, the microdroplets with a diameter of 290 µm were finally selected for subsequent experiments. The speeds of water and oil were 5 μL·min^−1^ and 60 μL·min^−1^, respectively, and the microdroplet generation speed was 50 drops per second.

In addition, considering the need for microdroplet stability and the high price of Pico-surf surfactants, the number of surfactants added was optimized. The collected microdroplets were sealed on grooved glass slides and regularly observed for damage in a constant temperature incubator at 30 °C. The breakage rate was calculated according to the area occupied by the broken droplet in the grooved slide to the total area of the groove (Fig. 1c). With the increase of surfactant addition, the stability of microdroplets was significantly improved (Fig. 1d). When the volume fraction of the surfactant was increased to 2%, the stability of the microdroplets could still reach more than 95% within 120 h, and higher concentration of the surfactant did not significantly improve the stability of the microdroplets. Therefore, surfactant addition with 2% was chosen for subsequent microdroplet generation.

### Validation and optimization of high-throughput screening method

**Fig. 2:**
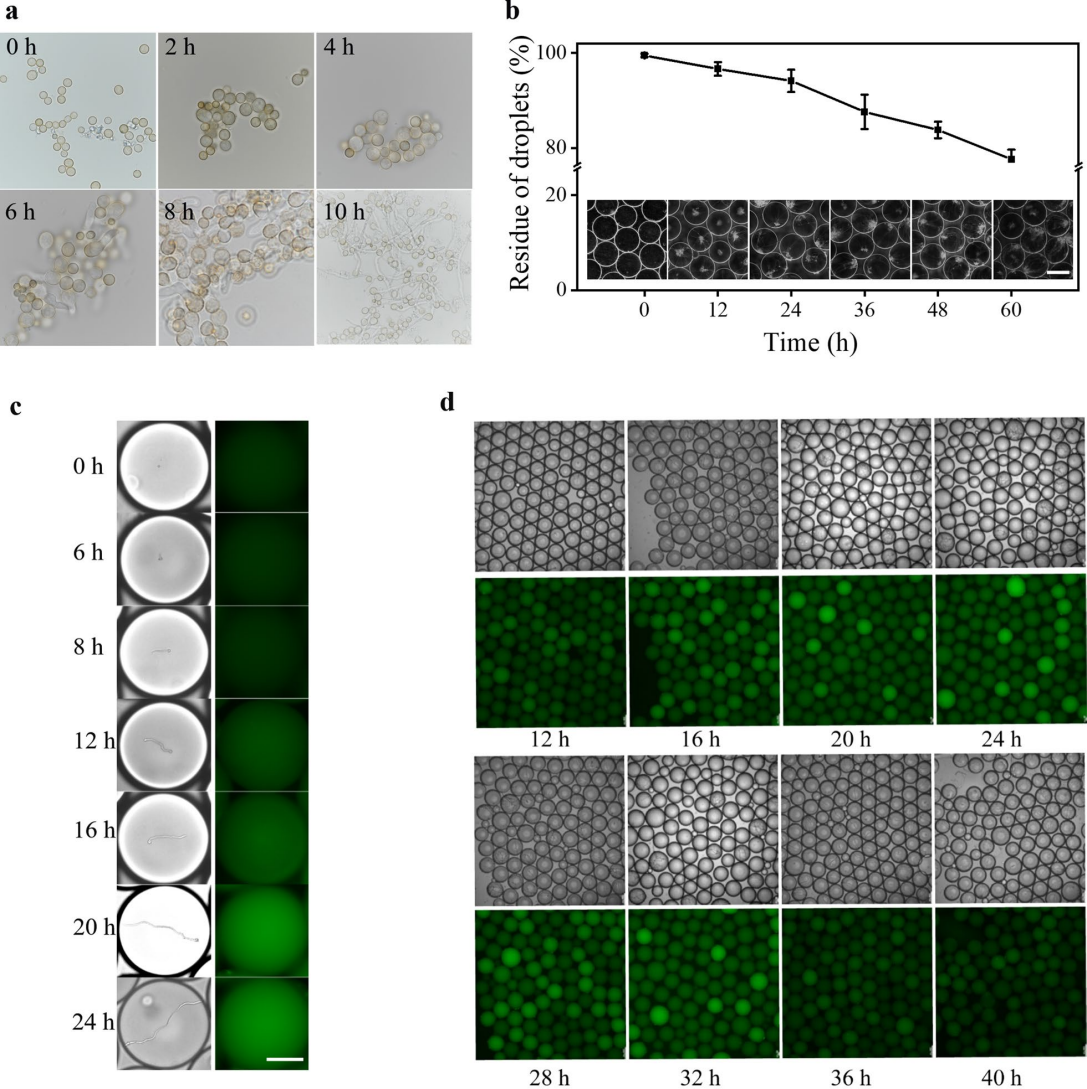
Validation of growth kinetics of *A. oryzae* in microdroplet. **a** Germination of spores in static culture. **b** Stability of droplets as hyphae grow in droplets. Scale bar: 250 μm. **c** Spore growth and fluorescence accumulation in droplets. Scale bar: 100 μm. **d** Fluorescence accumulation of droplets during mycelial growth

Compared with the cultural environment of shake flasks, the dissolved oxygen in the microdroplets is relatively poor. A simulation experiment was carried out to confirm that the spores of *A. oryzae* can germinate and grow smoothly. The spores of *A. oryzae* were inoculated into 1.5 mL centrifuge tubes filled with culture medium, and cultured at 30 °C, and the germination of the spores was observed by sampling regularly (Fig. 2a). The spores can normally germinate and grow within 10 h. To evaluate the stability of the hyphae-embedded microdroplets, each droplet was controlled to theoretically contain one spore (λ = 1) according to Poisson distribution for droplet entrapment [27]. Under this condition, the theoretical spore entrapment rate of the droplet was 63.2%. Some of the droplets contained multiple spores, which would bring greater pressure to the stability of the droplets. The breakage rate of the droplets was detected every 12 h (Fig. 2b). After being cultured for 12 h, apparent hyphae can be seen, indicating that the spores can germinate and rapidly grow in the droplets. For the stability of droplets, the droplet breakage rate after 24 h of culture was lower than 10% but increased significantly after being cultured for 36 h. That is because the length of the polar growing hyphae reached the length of the diameter and began to puncture droplets. After 36 h of incubation, the rate of droplet breakage was reduced, and the bending and branching of the hyphae could be seen. At this time, 80% of the intact droplets are still retained, indicating that the microdroplets have good stability.

To achieve single spore entrapment of microdroplets, each droplet was controlled to theoretically have only 0.2–0.3 (λ = 0.2–0.3) spores according to the Poisson distribution, although a large number of empty droplets formed in this way. Besides, DQ starch substrate was added to the aqueous phase at a final concentration of 50 µg·mL^−1^. Spore germination grew polar after 6 h of culture, consistent with the previous simulation results (Fig. 2c). With the extension of incubation time, the hyphae became longer and longer, and by 24 h, the length of the hyphae reached or exceeded the diameter of the microdroplet, which was the reason for the accelerated droplet breakage rate. However, a change in droplet fluorescence was observed by fluorescence microscopy at 12 h of incubation, indicating early accumulation of α-amylase. The fluorescence intensity of the droplets reached its peak after 20 h of incubation, and no further increase was observed after 24 h, potentially due to the degradation of fluorescent substances during prolonged incubation (Fig. 2c). To determine the change of fluorescence intensity in droplets during mycelial growth and to determine the optimal time for sorting, the microdroplets were observed continuously for 40 h (interval of 4 h). The same chip selected different regions for observation each time to avoid fluorescence quenching caused by multiple observations. The fluorescence of the droplets began to reach its peak after 20 h of incubation and remained stable from 20 to 32 h. Subsequently, the fluorescence intensity started to decline after 32 h (Fig. 2d). Therefore, “20 h” was chosen as the final sorting time.

**Fig. 3:**
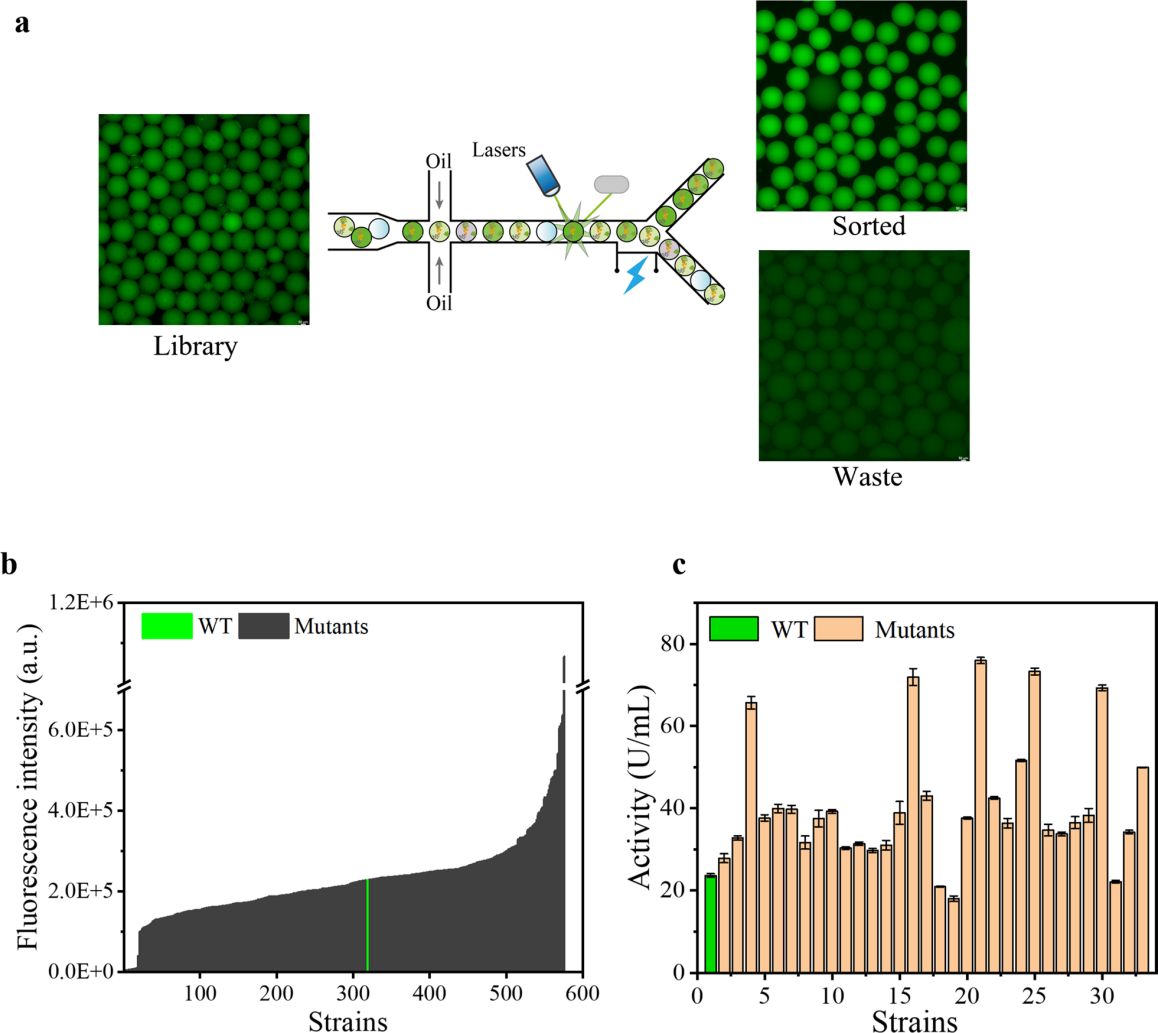
Validation of efficient screening effects. **a** Sorting effect of microfluidic system on microdroplets. **b** Results of the 48-deep-well plate primary screening of manually picked mutants. **c** Rescreening results of high-yielding strains in 48-deep-well plates

The mutation library was generated using the atmospheric and room temperature plasma (ARTP) technology [28, 29], and the mutagenesis conditions were optimized (Additional file 1: Fig. S2). The spore suspension mutation library was cultured for some time and then single spore embedding (λ = 0.2 ~ 0.3) was carried out [27]. Microfluidic sorting and enrichment were carried out after the microdroplets were cultured statically in a 30 °C incubator for 20 h. The flow rate of the separation phase was set at 1 µL·min^−1^, while the dispersion phase was maintained at 15 µL·min^−1^. The separation voltage used was 20 kHz, 1500 *Vpp*, 5 ms, resulting in a separation efficiency of 350 droplets per minute. Under the conditions of the sorting threshold of 8‰, approximately 100,000 droplets were successfully sorted. The statistics of the number of droplets were obtained based on the capture and counting of signals by a microscope. Fluorescence microscope observation of microdroplets before and after enrichment showed that microfluidics can effectively screen out highly fluorescent microdroplets (Fig. 3a). Then, the obtained spore suspension was diluted and coated on a PDA plate to obtain single colonies. Spores from every single colony were manually picked into a 48-deep-well plate for fermentation and culture for 48 h, and the fluorescence intensity was detected. 44.5% of the enriched mutant strains had higher enzyme activity than the wild type, indicating that the enrichment effect of the droplet microfluidic system was good (Fig. 3b). The top 5% of the fluorescence intensity (32 strains) were selected for shake-flask re-screening, and 29 strains had higher enzyme activity than the wild type, and the highest was 2.2 times higher than the wild type (Fig. 3c). The above results indicated that the high-throughput screening system based on droplet microfluidics technology can effectively screen high-efficiency secretion expression strains of *A. oryzae*.

### Optimization of the screening process to quickly obtain high-yielding strains

In the high-throughput screening process, the verification and separation of the spore suspension after microfluidic enrichment still required a lot of workforce and material resources, which seriously affected the efficiency of the entire screening process. In addition, some cases were observed, where the fluorescence intensity was abnormally high due to the high number of pellets. When manually picking *A. oryzae* spores, the same inoculum size cannot be controlled, which affects the accuracy of the screening results. Therefore, using FC sorted single spores into 96 shallow well plates for fermentation to eliminate the massive effect of different inoculum sizes. To avoid the reduction of the regeneration rate of the spore suspension by two consecutive power-on sortings, a plate expansion of the spores was carried out after microfluidic enrichment and then a new mixed spore suspension was obtained. Although plate expansion resulted in a spore suspension containing the same sample, it was also screened for genetic stability of the mutation and *A. oryzae* heterokaryon status.

**Fig. 4:**
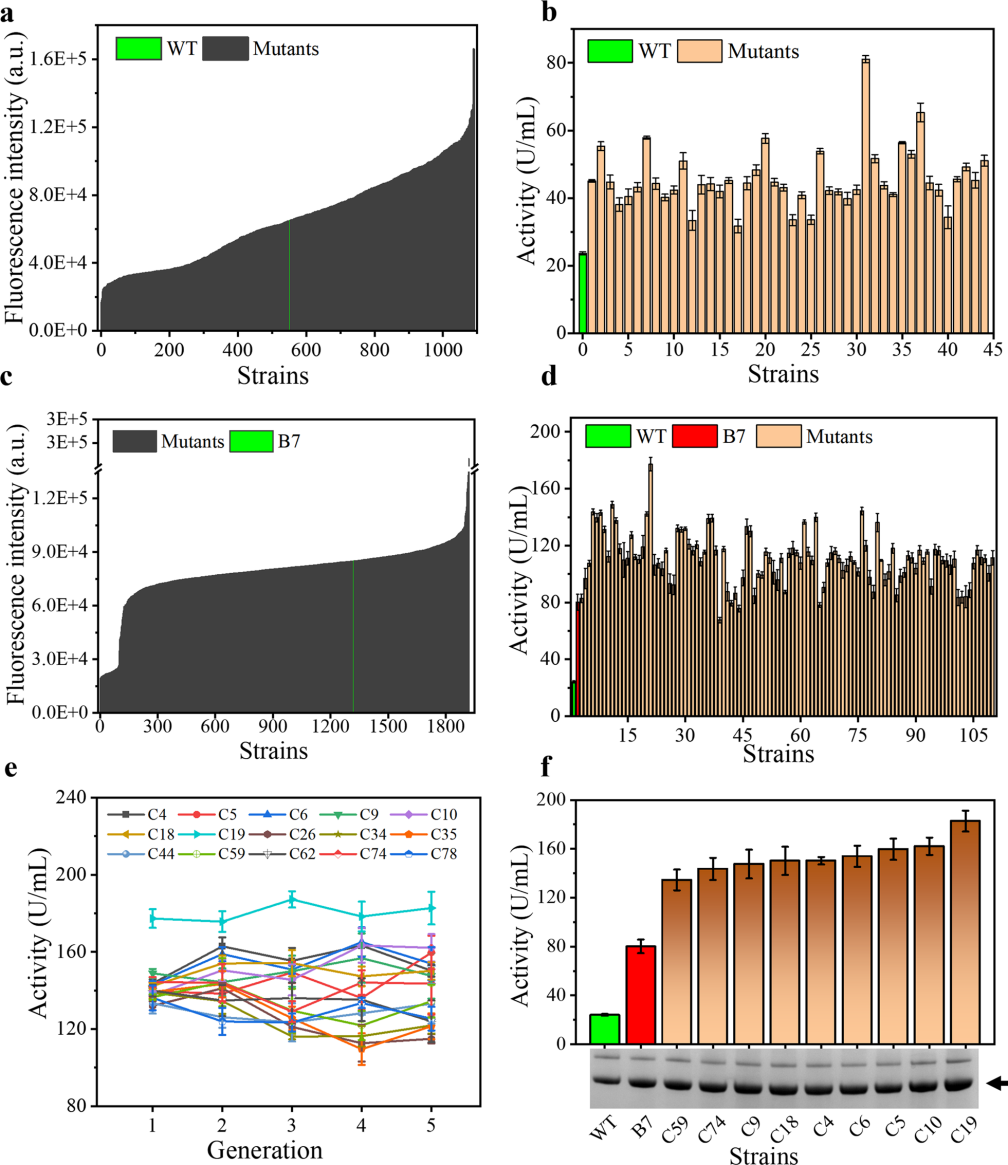
Screening of high α-amylase-producing *A. oryzae* strains.** a** Results of the 96-well microplate primary screening combined with FC. **b** Results of rescreening of high-yielding strains in 96 microplates with FC. **c** Preliminary screening results of iterative mutagenesis. **d** Shake-flask rescreening results of iterative mutagenesis.** e** Genetic stability of high-yielding strains. **f** Analysis of α-amylase expression level of high-yielding strains, arrows indicate α-amylase bands

Approximately 200,000 droplets were enriched using microfluidics, diluted and amplified on PDA plates, and a fresh spore suspension was recovered. Different plates were diluted to the same concentration and mixed in equal proportions. Then, single spores were sorted into 96-well microplates by FC, and the fluorescence intensity was detected after 24 h of fermentation (Fig. 4a). The sorted 1200 mutants still maintained an excellent enrichment effect. The high-yielding strains with the top 5% fluorescence intensity were selected for shake-flask rescreening. The results showed that 44 high-yielding strains were all higher than the wild-type strain (Fig. 4b), and the α-amylase activity of the high-yielding strain B7 was 2.4 times higher than that of the wild-type strain. Therefore, single spore sorting by FC not only improves the efficiency of high-throughput screening but also improves the accuracy of high-throughput screening.

A round of iterative mutagenesis based on the B7 strain was performed to obtain mutant strains with higher α-amylase activity. Approximately 250,000 droplets were enriched by microfluidics, and 2000 droplets were collected for subsequent culture, flow cytometry sorting, and shake-flask verification. The positive enrichment rate of microfluidics has decreased (Fig. 4c), but 97% of the rescreened strains selected at a ratio of 5% have higher α-amylase activity than B7 (Fig. 4d), and the highest strain C19 was 1.3 times higher than B7 and 6.6 times higher than the wild-type strain. For the enrichment of large samples, the positive enrichment rate can be increased by increasing the screening threshold.

The genetic stability experiment of 15 high-yielding strains obtained from the above screening showed that all high-yielding strains retained excellent characteristics (Fig. 4e). Nine mutants with high enzyme activity and B7 were selected for SDS–PAGE electrophoresis analysis. The expression of α-amylase in high-yield strains was significantly increased (Fig. 4f), which was attributed to the stable mutation in *A. oryzae*. The mechanisms underlying these mutations are of great interest to us.

### The selection of high-yield strains for genome resequencing

**Fig. 5:**
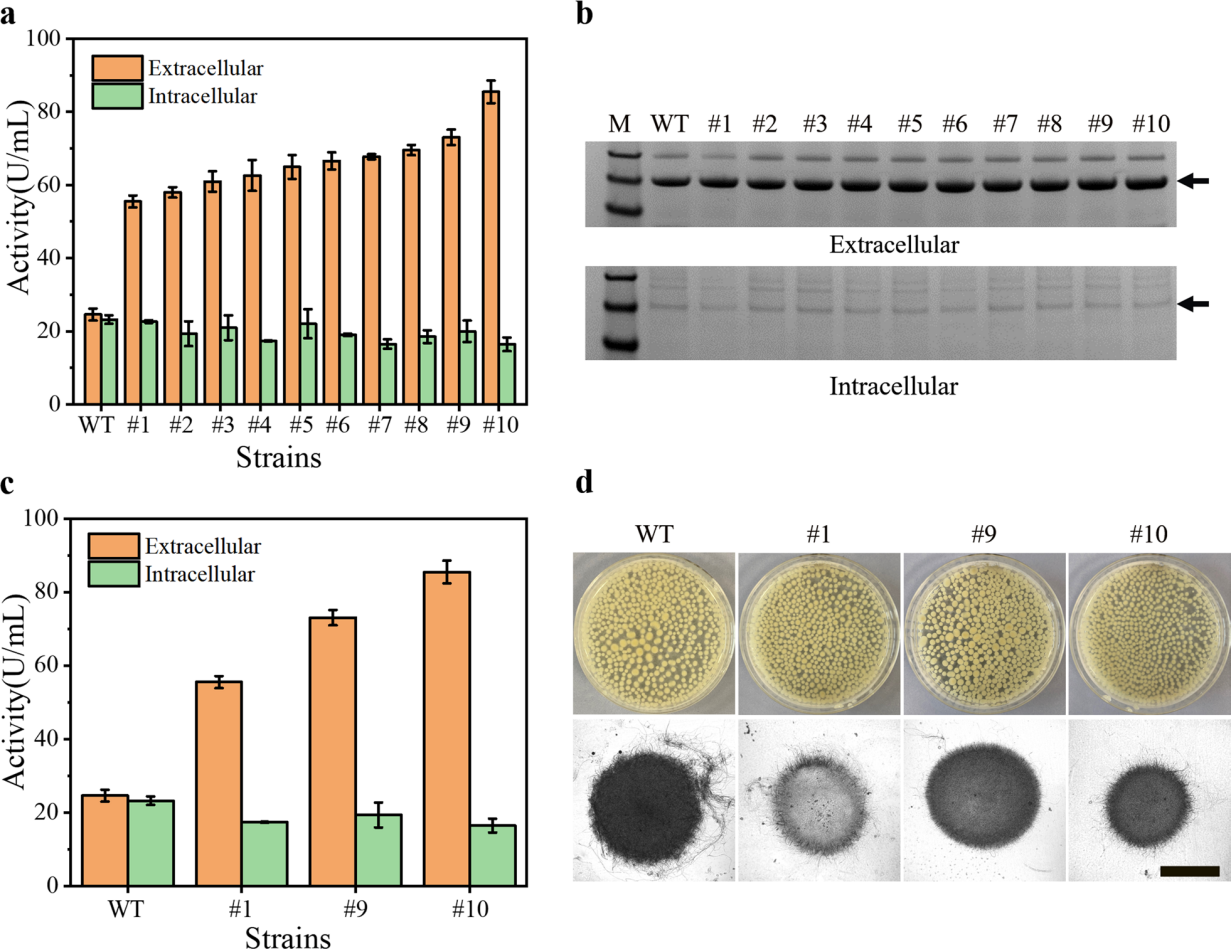
Protein expression and mycelium morphology of high-yielding strains. **a** Intracellular and extracellular enzyme activities of the high-yielding strains. **b** Intracellular and extracellular protein expression levels of the high-yielding strains, arrows indicate α-amylase bands. **c** Intracellular and extracellular enzyme activities of the selected high-yielding strains. **d** Mycelium morphology of the selected high-yielding strains. Scale bar: 500 µm

To explore the underlying mechanism of high-yielding α-amylase expression in *A. oryzae*, the intracellular and extracellular protein expression levels of some high-yielding strains were explored. These strains were obtained from the primary screening to reduce the complexity and crossover of the underlying mechanism (Fig. 4b). Compared with the wild strain, the extracellular enzyme activity of the high-yielding strain was significantly increased, and the intracellular enzyme activity had a slight downward trend (Fig. 5a). This indicated that the main reason for the high production of the mutant strain was the increased expression level of α-amylase, not entirely due to the translocation of intracellular proteins to the extracellular space (Fig. 5b). To more clearly reflect the differences among high-yielding strains, three strains with extracellular enzyme activities 1.26 times (#1), 1.97 times (#9) and 2.48 times (#10) higher than wild type were selected for comparison (Fig. 5c). Besides, the pellets of the three high-yielding strains were looser than those of the wild type, and the surface of the mycelial ball of #9 was smoother (Fig. 5d).

The whole-genome resequencing of #1, #9, and #10 strains was carried out to explore the high-yield mechanism of high-production α-amylase strains. After strict data evaluation and quality control, the data were compared and analyzed with the wild-type genome sequence. A total of 29 genes had hetero-sense mutations from these three strains, and the types of mutations included point mutations, frameshift mutations, and termination signals (Additional file 1: Table S3). To explore the influence mechanism of these 29 genes on the expression of α-amylase in *A. oryzae* to the greatest extent, Gene Ontology (GO), Kyoto Encyclopedia of Genes and Genomes (KEGG), and euKaryotic Ortholog Groups (KOG) enrichment were conducted. Functional annotations were performed on these genes, resulting in the annotation of 15 genes (Additional file 1: Table S3). However, according to *P* value and *Q* value, gene enrichment analysis showed that there was no significant enrichment of these mutant genes, which indicated that the effects of these mutations were diverse (Additional file 1: Tables S4, S5).

### Gene function analysis of high-yielding mutants

**Fig. 6:**
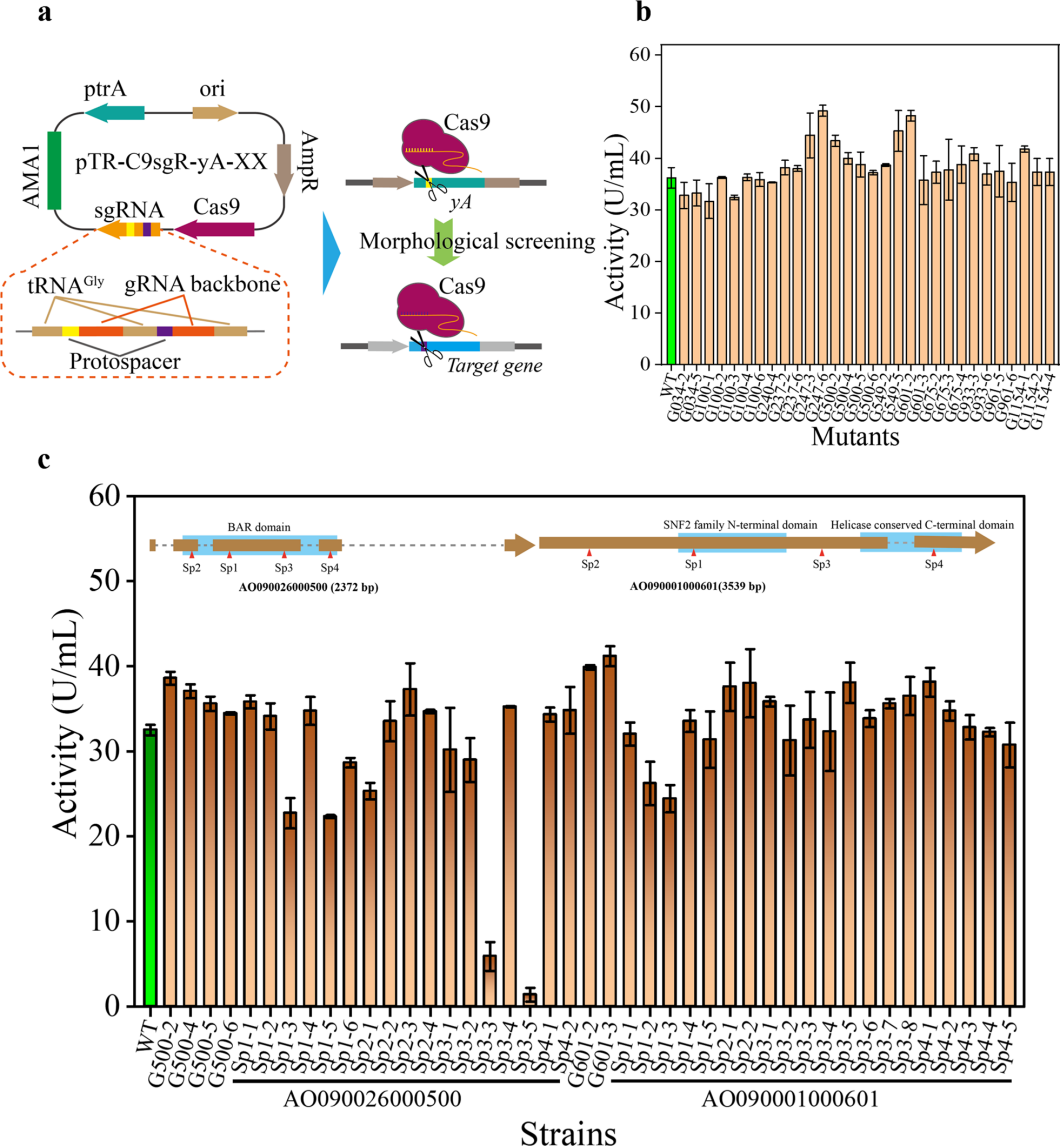
Effect of target gene mutation on expression of α-amylase in *A. oryzae*. **a** Schematic diagram of target gene editing based on the CRISPR–Cas9 system. **b** α-Amylase activity of different mutants related to the target genes. **c** Effect of mutations in different regions of AO090026000500 and AO090001000601 on the expression of α-amylase in *A. oryzae*. Strain SpX-N indicates that the mutation occurs near Spacer X, and N indicates the number of different mutant strains

Since the mutated 29 genes were all uncharacterized proteins, 15 functionally annotated genes were selected for editing experiments to explore their contributions to α-amylase expression (Fig. 6a). To simplify gene names, G plus the last three or four digits of the gene number are used. In addition, the following numbers indicate the different mutant strains. Different mutants of 12 genes were successfully obtained (Additional file 1: Fig. S3), and mutations in genes AO090026000500 and AO090001000601 exhibited noticeable effects on α-amylase expression (Fig. 6b). However, there was no obvious difference in the morphology of their mycelial balls (Additional file 1: Fig. S4), as the morphology of *A. oryzae* is mainly related to the composition and content of the cell wall, such as α-1,3-glucan and galactosaminogalactan [30, 31]. Besides, the differences in pellet diameter, surface smoothness, and compactness might be influenced by the cultivation conditions [32, 33]. Based on the National Center for Biotechnology Information (NCBI) exploration of the sequence and function of these two proteins, AO090026000500 was identified to encode a Bin/amphiphysin/Rvs (BAR) domain-containing protein, known to play important roles in a wide variety of cellular processes, such as organelle biogenesis, cell division, cell migration, secretion, and endocytosis, by serving as central regulators of dynamic membrane remodelling [34, 35]. AO090001000601 encodes a protein containing an N-terminal domain of the SNF2 family, which is involved in transcriptional regulation and DNA repair [36]. Hence, the mutation of AO090026000500 may affect the integrity and stability of the membrane structure, thereby influencing the translation and transport process of α-amylase, while AO090001000601 may impact the transcription of related genes to affect the expression level of α-amylase.

The gene AO090026000500 has a full length of 2372 bp, contains 4 introns, and encodes 322 amino acids, of which amino acids 44–287 are BAR domains (Fig. 6c). The gene AO090001000601 is 3539 bp in length, including an intron, and encodes 1157 amino acids. Among them, amino acids 354–706 are the SNF2 family domain, and amino acids 919–1030 are the conserved C-terminal domain of the helicase. To confirm the influence of the two genes on the expression of α-amylase in *A. oryzae* and obtain a higher response effect to provide materials for the subsequent analysis of their regulatory mechanism, different regions of the two genes were edited based on the CRISPR/Cas9 system. After sequencing verification, 17 and 20 different mutants were obtained for the two genes, respectively (Additional file 1: Fig. S5). The mutations in the two genes indeed have an impact on the expression of α-amylase in *A. oryzae*. The effects generated by the mutations in the predicted functional domains are more pronounced, while they do not influence the morphology of *A. oryzae* pellets (Fig. 6c, Additional file 1: Fig. S6). In gene AO090026000500, spacer1 is located within the membrane-binding sequence, amphipathic α-helix, of the BAR domains. Therefore, mutations in this region might affect the protein’s binding to the membrane, subsequently influencing cellular processes (Additional file 1: Fig. S7a). On the other hand, mutations in the spacer3 region have the most significant impact on α-amylase expression, suggesting its crucial role in stabilizing the formation of dimers and functional activity. Similarly, the mutations within gene AO090001000601 that have a significant impact occur in the flexible loop structure of the SNF2 domain, which illustrates the importance of this domain for protein function (Additional file 1: Fig. S7b).

In the process of single gene editing and screening, 21 and 22 mutant strains of gene AO090026000500 and gene AO090001000601 were obtained, respectively. Compared with the wild type, the distribution of extracellular enzyme activity of the mutant strain of the gene AO090026000500 ranged from 4.3% to 118.8%, and that of the gene AO090001000601 was 75.1–126.8%, which indicated that these two genes had a significant effect on the expression of α-amylase. However, although some mutant strains with increased expression levels of α-amylase were obtained, the expression levels of the selected strains were far from reaching the expression level of the selected strains, which indicated that these mutated genes may have mutual promoting or additive effects [37, 38]. Multigene combinatorial mutation screening may be more effective in increasing the expression level of α-amylase. Besides, the above results also indicated that there may be more efficient regulatory mechanisms in other unverified genes that need to be mined and verified.

## Conclusion

In this study, a high-throughput screening method based on a microfluidic system and FC was established for *A. oryzae*, which can precisely complete the high-efficiency screening of 450,000 mutants within 2 weeks. After a round of iterative mutagenesis screening, the α-amylase activity of *A. oryzae* increased by 6.6 times, reflecting the compatibility of this screening method for the polar growth characteristics of mycelia. Based on genome resequencing and CRISPR editing, the related genes affecting the production of α-amylase in *A. oryzae* were explored and analyzed, and two genes were found to affect the expression of α-amylase through membrane-associated multicellular processes and the transcription of related genes. This developed strategy is also suitable for high-throughput screening of other filamentous fungi and *Streptomyces*. Besides, the strains with different excellent characteristics obtained by efficient screening can not only meet the needs of industrial production but also provide materials for the construction of the *A. oryzae* expression system and the analysis of genetic and regulatory mechanisms.

## Materials and methods

### Strain, media, and culture conditions

*Aspergillus oryzae* RIB40 (ATCC42149) was used as the control strain for high-throughput screening. Both the activation and sporulation of the strains were cultured on potato dextrose agar (PDA) plates at 30 °C for 3 days. The medium used for spore germination growth in microdroplets, 48-well deep plate, and 96-well microplates was Czapek–Dox Medium (CD) containing sucrose 2%, NaNO_3_ 0.3%, K_2_HPO_4_ 0.1%, KCl 0.05%, MgSO_4_·7H_2_O 0.05%, FeSO_4_ 0.001%. A 250 mL Erlenmeyer flask, with a liquid volume of 60 mL fermentation medium, was aseptically inoculated with 1.5 mL of a spore suspension (10^7^ spores·mL^−1^) and subjected to agitation at 220 rpm and 30 °C for 72 h to facilitate the fermentation of amylase. The formula of fermentation medium includes dextrin 2%, peptone 0.5%, yeast extract 0.1%, NaNO_3_ 0.1%, KH_2_PO_4_ 0.05%, MgSO_4_·7H_2_O 0.05%, FeSO_4_·7H_2_O 0.001%.

### Droplet generation optimization and microfluidic screening

The oil and water phases were drawn into 1 mL sterile syringes, mounted in a micro syringe pump (Baoding Longer Precision Pump Co., Ltd. China), and connected to the chip with a catheter. The flow rates of the water and oil phases are controlled within a range to generate droplets. To achieve stable and uniform microdroplet generation, the optimization process initially focused on selecting the appropriate type of oil phase. Three commonly used oils in droplet microfluidics, including FC40, Novec7500, and Bio-Rad, were evaluated for their suitability. Then, 5, 10, and 20 µ·min^−1^ for the water phase flow rate and 60, 100, and 140 µL·min^−1^ for the oil phase were chosen to optimize microdroplet sizes. In addition, different concentrations of surfactants (0.1%, 0.5%, 1%, 2%, and 5%) were optimized to balance droplet stability and the high price of Pico-surf surfactants.

The generated droplets were collected into a 1 mL sterile syringe, sealed, and placed in a constant temperature incubator at 30 °C for incubation. The oil phase was Pico-surf™ 1 (2% (w/w) in Novec™ 7500), and the water phase was a CD medium containing a specific final concentration of spores (according to Poisson distribution, control 0.2–0.3 spores per droplet, i.e., λ = 0.2–0.3) and 50 ug·mL^−1^ of DQ starch substrate (BODIPY fluorescein-conjugated starch, Thermo Fisher Scientific, Shanghai, China), and the diameter of the resulting droplets was controlled at 200–350 µm. During the screening, the syringes containing the oil phase and the droplets were installed into the micro-injection pump, respectively. The outlet of the syringe was connected to the corresponding inlet of the microfluidic droplet sorting chip with a catheter, and the flow rate of the oil phase was set as 10–15 µL·mL^−1^ and the flow rate of the droplets as 1–5 µL·mL^−1^. The blue laser (488 nm) was aimed at the detection point of the screening chip, the green fluorescence signal (520 nm) of each droplet was collected, and the droplet with the highest fluorescence intensity of about 8‰ was selected. The selection of the threshold was achieved by controlling the ratio of the number of droplets that trigger the sorting signal to the total number of droplets based on signal strength during pre-screening. The AC field pulses (20 kHz, 1500 *Vpp*, 5 ms) were set to make these droplets flow to the droplet collection outlet of the screening chip and apply the collected droplets to the PDA plate [39].

### Microplate screening of enriched samples

The PDA plate was eluted with 0.5% Tween 80 solution, and the residual hyphae were removed by double-layer Miracloth filtration to obtain a clean mixed spore suspension. Single spores were injected into a 96-well microplate containing 60 µL per well of CD medium by FC and cultured at 30 °C with shaking for 24 h. The supernatant was collected by centrifugation (9000 rpm, 5 min), diluted, and reacted with DQ starch substrate with a final 50 ug·mL^−1^ concentration, and then the green fluorescence signal (Ex = 488 nm/Em = 520 nm) was detected by a microplate reader, and high-yielding strains were selected. BODIPY fluorescein is embedded in the DQ starch substrate skeleton. The fluorescein is inside the starch granules and cannot generate fluorescence in the initial state. When it is hydrolyzed by α-amylase, the cleavage of the starch chain will expose the fluorescein in it, and then the enzyme activity is detected by the fluorescence intensity using the correlation between the starch hydrolysis rate and the enzyme activity [23].

### Shake-flask fermentation

The high-yielding strains screened by the microplate were purified, isolated, activated, and eluted after maturation to obtain a spore suspension (10^7^ spores·mL^−1^). Then, 2.5% of the inoculum was inoculated into the fermentation medium, and it was fermented at 30 °C for 72 h. Among them, the rotation speed of the shaker was set to 200 rpm for the first 12 h, and 220 rpm after 12 h to minimize the effect of adherent growth on fermentation.

### α-Amylase activity assay

The α-amylase activity in the supernatant of the fermentation broth was determined by the modified 3,5-dinitrosalicylic acid (DNS) method [40]. The detailed steps were as follows: 2% starch solution was prepared with pH 5.6 citric acid–sodium citrate buffer as the enzyme reaction substrate. 900 μL of the pre-warmed substrate was accurately reacted with 100 μL of appropriately diluted enzyme solution at 50 °C for 10 min, and then 2 mL of DNS reagent was added to stop the reaction. The colour was developed in a boiling water bath for 10 min, the volume was made up to 25 mL with deionized water, and the absorbance at 520 nm was measured. The inactivated enzyme was used as a control. α-Amylase activity in the fermentation broth supernatant was calculated from a standard curve prepared with glucose. Enzyme activity is defined as under certain conditions (50 °C, pH 5.6), the amount of enzyme that generates 1 mg of glucose in 1 min of reaction is one unit of enzyme activity U.

### Genetic stability analysis of high-yield strains

The selected high-yielding strains were continuously sub-cultured 5 times, and the enzyme activity of α-amylase in each generation was evaluated by shaking flask fermentation. The cultivation conditions involved shaking 250 mL flasks with a liquid volume of 60 mL at 30 ℃ and 220 rpm for 3 days. Assessing the genetic stability of high-yield strains based on the α-amylase enzyme activity.

### Disruption of *A. oryzae* cells

Take a certain weight of *A. oryzae* mycelium balls in a mortar, and use liquid nitrogen to completely solidify the mycelial balls. Then, the mycelium balls were ground into powder and all were transferred into 5 mL centrifuge tubes, and resuspended with PBS buffer of pH 7.2. The supernatant was collected by centrifugation (12,000 rpm, 10 min) to obtain the intracellular soluble crude enzyme solution [41].

### Genome resequencing and gene editing by CRISPR–Cas9

The genome resequencing work was entrusted to Sangon Biotech (Shanghai) Co., Ltd. for implementation. Raw data were mapped by Illumina Hiseq™ [42] and evaluated using FastQC [43]. Trimmomatic was used for data processing. The target gene selected after analysis was edited and quickly screened using the CRISPR–Cas9 system based on the morphological gene *yA* as an indicator [44]. Mutants were selected by *A. oryzae* colony PCR and sequencing for subsequent experimental verification [44]. The primers and plasmids required for the construction of the CRISPR–Cas9 system are listed in Additional file 1: Tables S1 and S2.

### Supplementary Information

**Additional file 1: Figure S1.** Optimization of oils for microdroplet generation. **a** Droplet generation using Bio-Rad. Scale bar: 200 µm; **b** Droplet generation using Novec7500. Scale bar: 200 µm. **Figure S2.** Lethality curve of ARTP mutagenesis of *A. oryzae* spores. Other mutagenesis conditions were set as follows: the distance between the slide and the jet outlet of the plasma generator was 2 mm; the ventilation volume was 10 SLM; and the irradiation power was 120 W. **Figure S3.** Genetic information of mutations in relevant target genes. The sequence with green shading is original sequence. **Figure S4.** Pellet morphology of mutants of AO090026000500 and AO090001000601. Scale bar: 500 µm. **Figure S5.** Genetic information of different mutants of AO090026000500 and AO090001000601. The sequence with green shading is original sequence. **Figure S6.** Pellet morphology of mutants of AO090026000500 and AO090001000601. **Figure S7.** Protein structure prediction of AO090026000500 and AO090001000601 based on AlphaFold. AlphaFold produces a per-residue confidence score (pLDDT) between 0 and 100. Some regions below 50 pLDDT may be unstructured in isolation. **Table S1.** Main primers used in this study. **Table S2.** Main plasmids used in this study. **Table S3.** Mutations involved in high-yielding strains. fs means frameshift, * means Stop gained, del means deletion, Ter means stop codon, and ext* means stop lost. The selected genes for validation are marked in orange shading. **Table S4.** Result of Gene Ontology (GO) enrichment. **Table S5.** Result of euKaryotic Ortholog Groups (KOG) enrichment.

## Data Availability

Not applicable.
