# Supplementary material for: High-throughput droplet microfluidics screening and genome sequencing analysis for improved amylase-producing Aspergillus oryzae
Source: Biotechnol Biofuels Bioprod. 2023 Nov 29;16:185. doi: 10.1186/s13068-023-02437-6 (PMC10685594; doi:10.1186/s13068-023-02437-6)
Supplement: Supplementary file 1 — Additional file 1: Figure S1. Optimization of oils for microdroplet generation. a Droplet generation using Bio-Rad. Scale bar: 200 µm; b Droplet generation using Novec7500. Scale bar: 200 µm. Figure S2. Lethality curve of ARTP mutagenesis of A. oryzae spores. Other mutagenesis conditions were set as follows: the distance between the slide and the jet outlet of the plasma generator was 2 mm; the ventilation volume was 10 SLM; and the irradiation power was 120 W. Figure S3. Genetic information of mutations in relevant target genes. The sequence with green shading is original sequence. Figure S4. Pellet morphology of mutants of AO090026000500 and AO090001000601. Scale bar: 500 µm. Figure S5. Genetic information of different mutants of AO090026000500 and AO090001000601. The sequence with green shading is original sequence. Figure S6. Pellet morphology of mutants of AO090026000500 and AO090001000601. Figure S7. Protein structure prediction of AO090026000500 and AO090001000601 based on AlphaFold. AlphaFold produces a per-residue confidence score (pLDDT) between 0 and 100. Some regions below 50 pLDDT may be unstructured in isolation. Table S1. Main primers used in this study. Table S2. Main plasmids used in this study. Table S3. Mutations involved in high-yielding strains. fs means frameshift, * means Stop gained, del means deletion, Ter means stop codon, and ext* means stop lost. The selected genes for validation are marked in orange shading. Table S4. Result of Gene Ontology (GO) enrichment. Table S5. Result of euKaryotic Ortholog Groups (KOG) enrichment. [file 13068_2023_2437_MOESM1_ESM.docx]

**High-throughput droplet microfluidics screening and genome sequencing analysis for improved amylase-producing *Aspergillus oryzae***

Qinghua Li^1,2^, Jinchang Lu^1,2^, Jingya Liu^1,2^, Jianghua Li^1,3^, Guoqiang Zhang^1,2^*, Guocheng Du^1,3,4^, Jian Chen^1,2,3,4^

1. Science Center for Future Foods, Jiangnan University, 1800 Lihu Road, Wuxi, Jiangsu 214122, China

2. National Engineering Research Center for Cereal Fermentation and Food Biomanufacturing, Jiangnan University, 1800 Lihu Road, Wuxi, Jiangsu 214122, China

3. School of Biotechnology and Key Laboratory of Industrial Biotechnology, Ministry of Education, Jiangnan University, 1800 Lihu Road, Wuxi, Jiangsu 214122, China

4. The Key Laboratory of Carbohydrate Chemistry and Biotechnology, Ministry of Education, Jiangnan University, 1800 Lihu Road, Wuxi, Jiangsu 214122, China

* Corresponding authors: gqzhang@jiangnan.edu.cn

Supplementary data:

This file contains 7 figures and 5 tables.


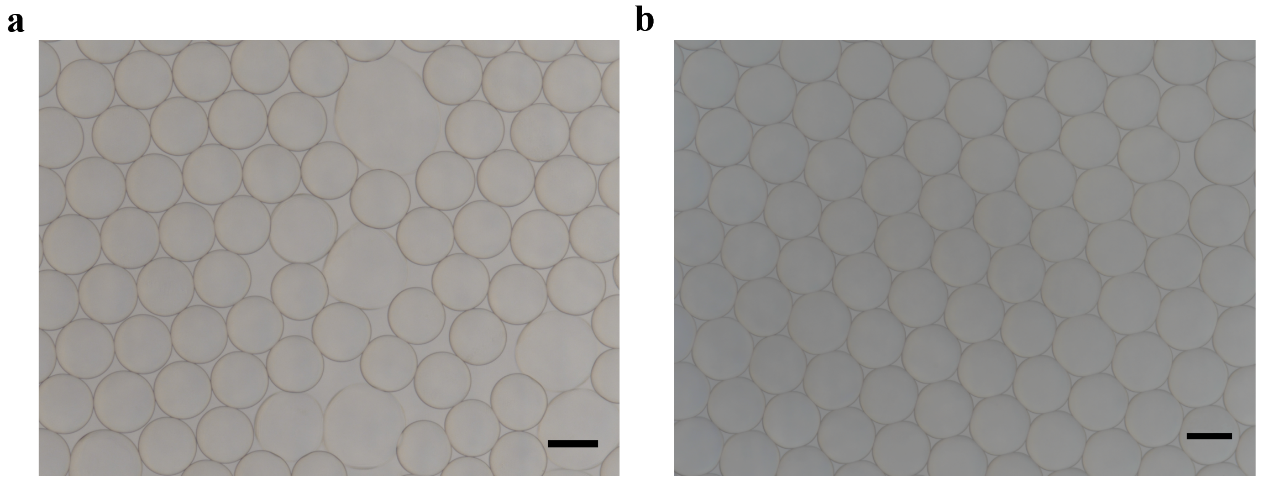


Fig. S1 Optimization of oils for microdroplet generation. **a** Droplet generation using Bio-Rad. Scale bar: 200 µm; **b** Droplet generation using Novec7500. Scale bar: 200 µm.


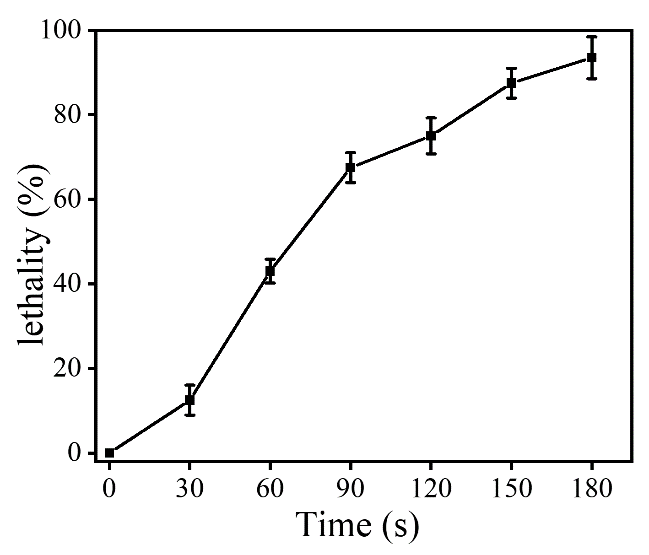


Fig. S2 The Lethality curve of ARTP mutagenesis of *A. oryzae* spores. Other mutagenesis conditions were set as follows: the distance between the slide and the jet outlet of the plasma generator was 2 mm; the ventilation volume was 10 SLM; and the irradiation power was 120 W.


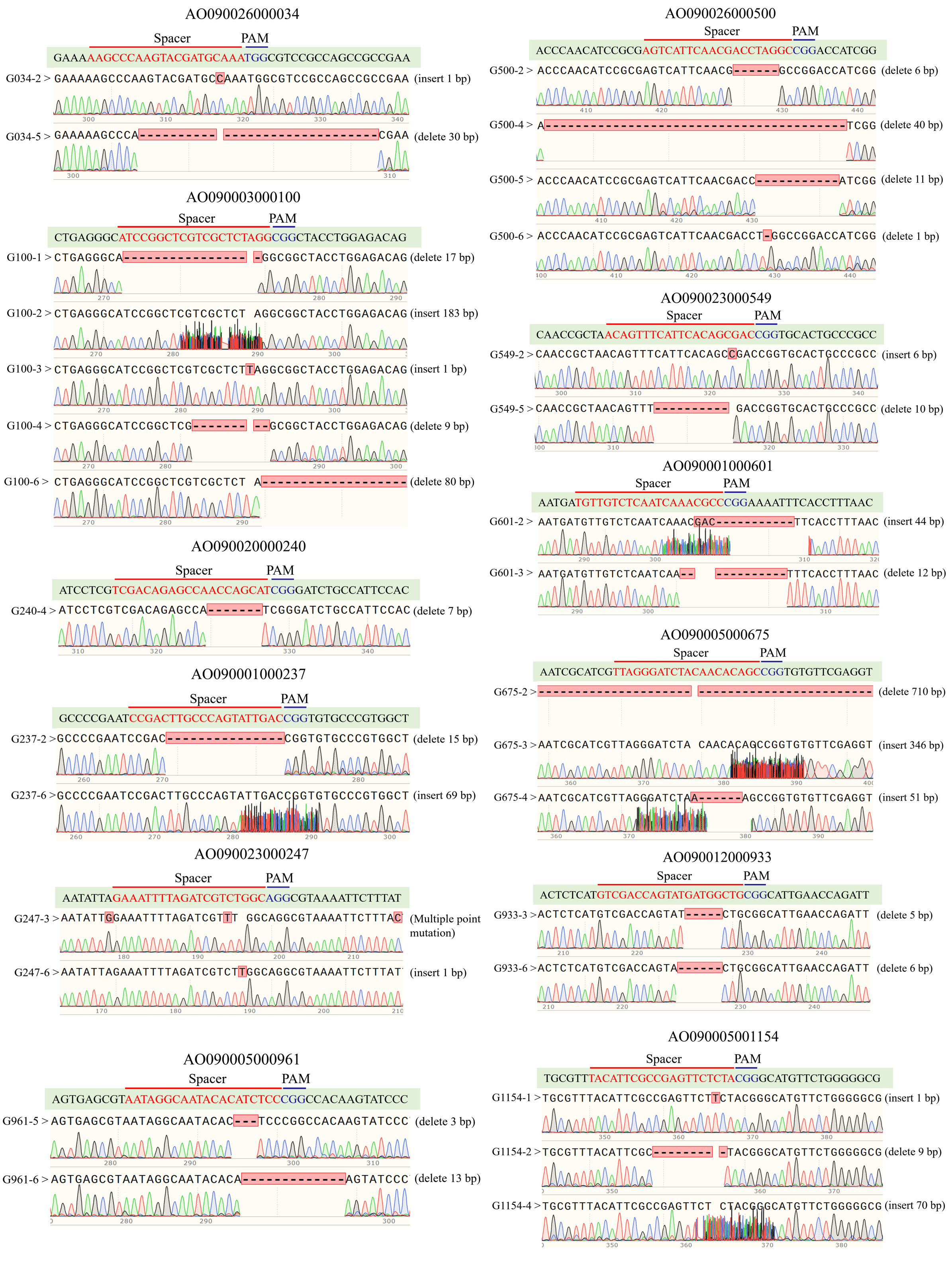


Fig. S3 The genetic information of mutations in relevant target genes. The sequence with green shading is original sequence.


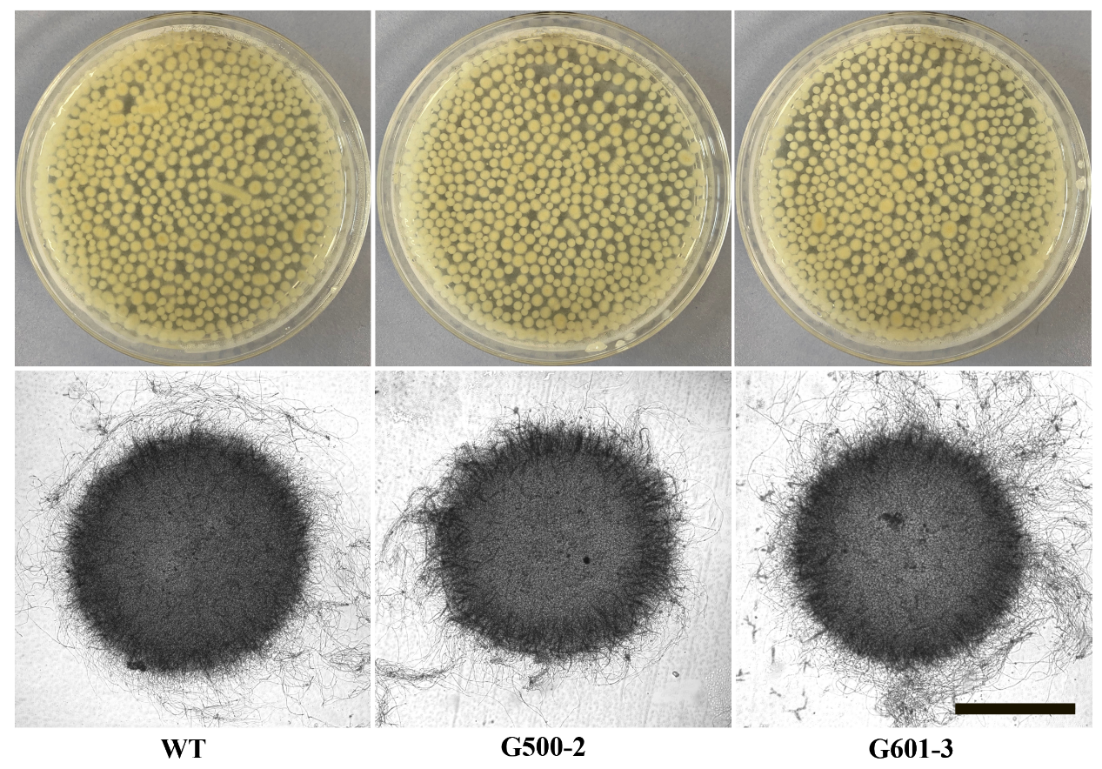


Fig. S4 The pellet morphology of mutants of AO090026000500 and AO090001000601. Scale bar: 500 µm.


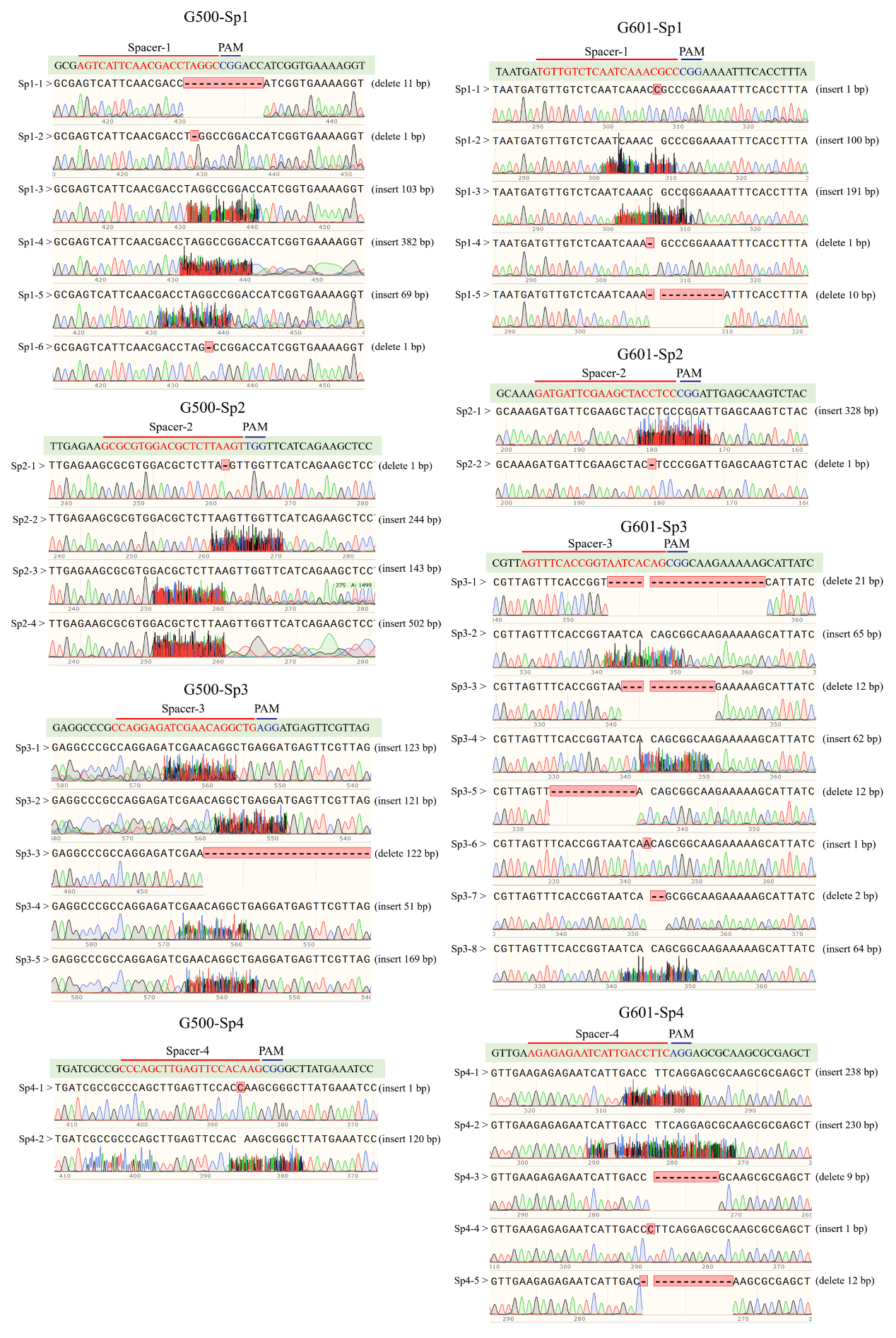


Fig. S5 The genetic information of different mutants of AO090026000500 and AO090001000601. The sequence with green shading is original sequence.


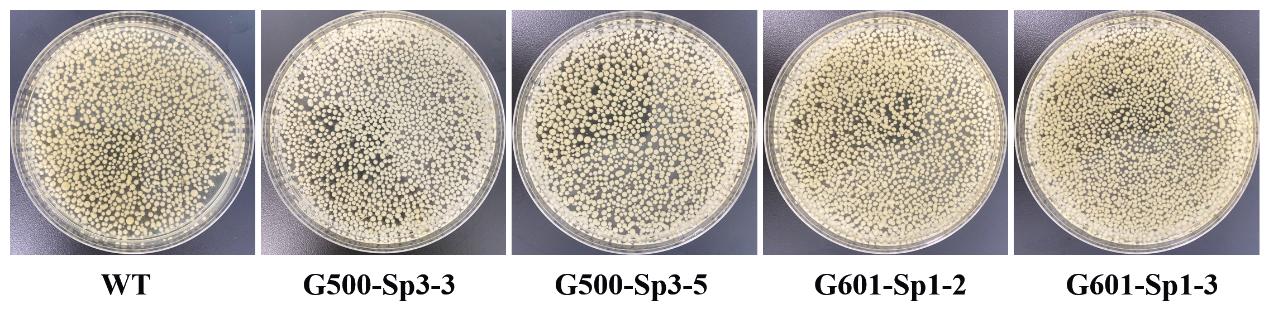


Fig. S6 The pellet morphology of mutants of AO090026000500 and AO090001000601.


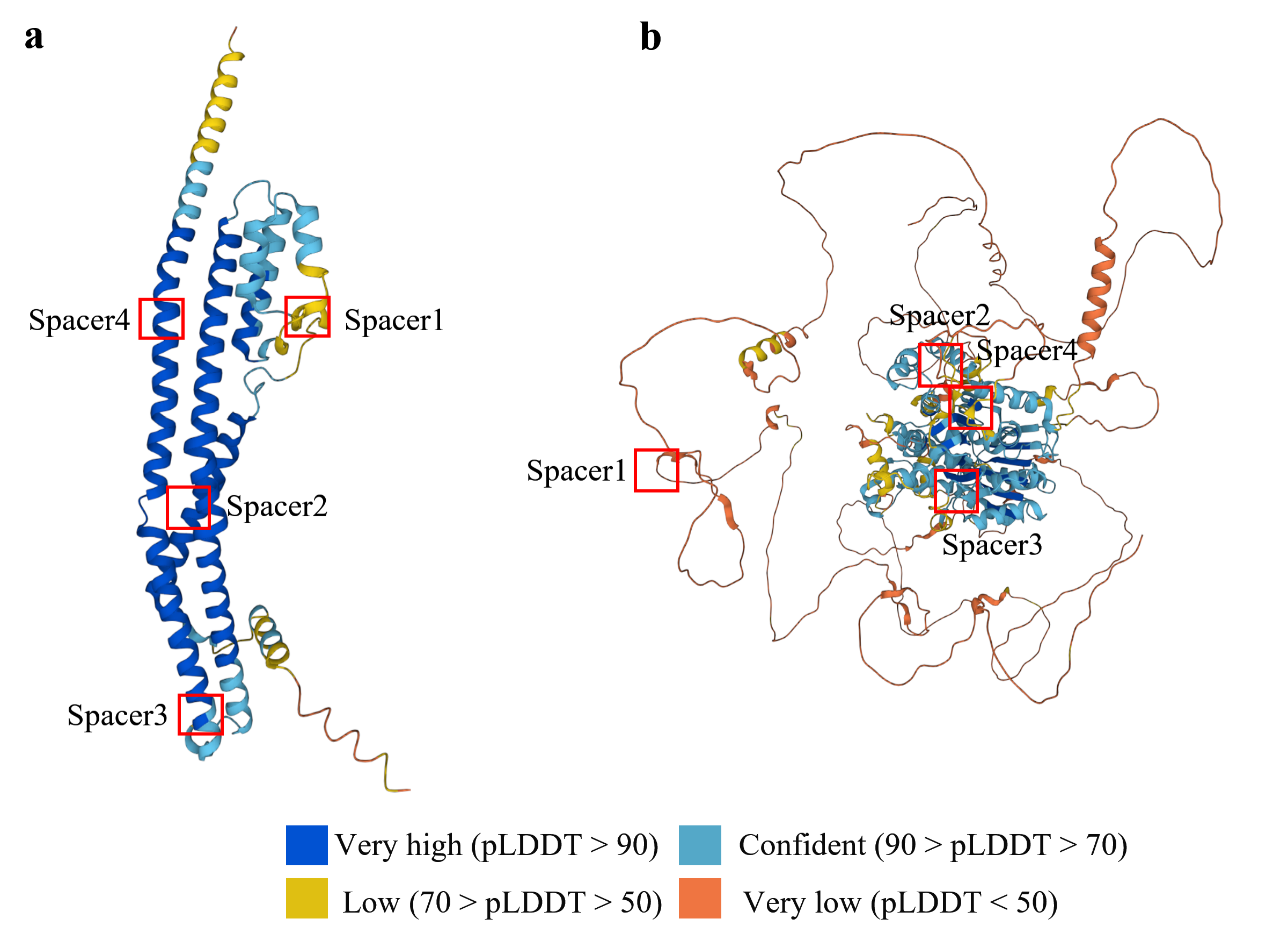


Fig. S7 The protein structure prediction of AO090026000500 and AO090001000601 based on AlphaFold. AlphaFold produces a per-residue confidence score (pLDDT) between 0 and 100. Some regions below 50 pLDDT may be unstructured in isolation.

Table S1. The main primers used in this study.

| **Primers** | **Sequence (5’ to 3’)** |
| --- | --- |
| G237-F | ccagtattgacGTTTTAGAGCTAGAAATAGCAAG |
| G237-R | ggcaagtcggTGCATCATCCGTGAATCG |
| G261-F | ggcttcagtcGTTTTAGAGCTAGAAATAGCAAG |
| G261-R | tcggtgtggaTGCATCATCCGTGAATCG |
| G364-F | catccaagaaGTTTTAGAGCTAGAAATAGCAAG |
| G364-R | cggtcgaggtTGCATCATCCGTGAATCG |
| G601-F1 | atcaaacgccGTTTTAGAGCTAGAAATAGCAAG |
| G601-R1 | tgagacaacaTGCATCATCCGTGAATCG |
| G100-F | tcgctctaggGTTTTAGAGCTAGAAATAGCAAG |
| G100-R | cgagccggatTGCATCATCCGTGAATCG |
| G675-F | acaacacagcGTTTTAGAGCTAGAAATAGCAAG |
| G675-R | agatccctaaTGCATCATCCGTGAATCG |
| G961-F | acacatctccGTTTTAGAGCTAGAAATAGCAAG |
| G961-R | attgcctattTGCATCATCCGTGAATCG |
| G1154-F | gagttctctaGTTTTAGAGCTAGAAATAGCAAG |
| G1154-R | ggcgaatgtaTGCATCATCCGTGAATCG |
| G933-F | atgatggctgGTTTTAGAGCTAGAAATAGCAAG |
| G933-R | actggtcgacTGCATCATCCGTGAATCG |
| G240-F | caaccagcatGTTTTAGAGCTAGAAATAGCAAG |
| G240-R | gctctgtcgaTGCATCATCCGTGAATCG |
| G247-F | atcgtctggcGTTTTAGAGCTAGAAATAGCAAG |
| G247-R | ctaaaatttcTGCATCATCCGTGAATCG |
| G549-F | tcacagcgacGTTTTAGAGCTAGAAATAGCAAG |
| G549-R | atgaaactgtTGCATCATCCGTGAATCG |
| G034-F | acgatgcaaaGTTTTAGAGCTAGAAATAGCAAG |
| G034-R | acttgggcttTGCATCATCCGTGAATCG |
| G500-F1 | cgacctaggcGTTTTAGAGCTAGAAATAGCAAG |
| G500-R1 | ttgaatgactTGCATCATCCGTGAATCG |
| G001-F | tgctatcatcGTTTTAGAGCTAGAAATAGCAAG |
| G001-R | accggccatcTGCATCATCCGTGAATCG |
| HiFi-const-F | TGTGGATAACCGTATTACCGCCTTTGAGTGAGCTGATTTAATTA  AGCCGACATAGCTGTTTCCGCTGAGG |
| HiFi-const-R | GCTACAGGGCGCGTACTATGGTTGCTTTGACGTATGCGTCCCTGGGAG |
| G500-F2 | gctcttaagtGTTTTAGAGCTAGAAATAGCAAG |
| G500-R2 | gtccacgcgcTGCATCATCCGTGAATCG |
| G500-F3 | gaacaggctgGTTTTAGAGCTAGAAATAGCAAG |
| G500-R3 | gatctcctggTGCATCATCCGTGAATCG |
| G500-F4 | gttccacaagGTTTTAGAGCTAGAAATAGCAAG |
| G500-R4 | tcaagctgggTGCATCATCCGTGAATCG |
| G601-F2 | agctacctccGTTTTAGAGCTAGAAATAGCAAG |
| G601-R2 | tcgaatcatcTGCATCATCCGTGAATCG |
| G601-F3 | gtaatcacagGTTTTAGAGCTAGAAATAGCAAG |
| G601-R3 | cggtgaaactTGCATCATCCGTGAATCG |
| G601-F4 | attgaccttcGTTTTAGAGCTAGAAATAGCAAG |
| G601-R4 | gattctctctTGCATCATCCGTGAATCG |

Table S2. The main plasmids used in this study.

| **Plasmids** | **Source** |
| --- | --- |
| pPTR II | Takara Bio Inc. |
| pC9sgR-Model | This study constructed |
| pC9sgR-yA-G1154 | This study constructed |
| pC9sgR-yA-G961 | This study constructed |
| pC9sgR-yA-G675 | This study constructed |
| pC9sgR-yA-G261 | This study constructed |
| pC9sgR-yA-G364 | This study constructed |
| pC9sgR-yA-G601 | This study constructed |
| pC9sgR-yA-G100 | This study constructed |
| pC9sgR-yA-G549 | This study constructed |
| pC9sgR-yA-G500 | This study constructed |
| pC9sgR-yA-G034 | This study constructed |
| pC9sgR-yA-G933 | This study constructed |
| pC9sgR-yA-G240 | This study constructed |
| pC9sgR-yA-G001 | This study constructed |
| pC9sgR-yA-G237 | This study constructed |
| pC9sgR-yA-G247 | This study constructed |
| pTR-C9sgR-yA-G1154 | This study constructed |
| pTR-C9sgR-yA-G961 | This study constructed |
| pTR-C9sgR-yA-G675 | This study constructed |
| pTR-C9sgR-yA-G261 | This study constructed |
| pTR-C9sgR-yA-G364 | This study constructed |
| pTR-C9sgR-yA-G601 | This study constructed |
| pTR-C9sgR-yA-G100 | This study constructed |
| pTR-C9sgR-yA-G549 | This study constructed |
| pTR-C9sgR-yA-G500 | This study constructed |
| pTR-C9sgR-yA-G034 | This study constructed |
| pTR-C9sgR-yA-G933 | This study constructed |
| pTR-C9sgR-yA-G240 | This study constructed |
| pTR-C9sgR-yA-G001 | This study constructed |
| pTR-C9sgR-yA-G237 | This study constructed |
| pTR-C9sgR-yA-G247 | This study constructed |
| pC9sgR-yA-G500-2 | This study constructed |
| pC9sgR-yA-G500-3 | This study constructed |
| pC9sgR-yA-G500-4 | This study constructed |
| pC9sgR-yA-G601-2 | This study constructed |
| pC9sgR-yA-G601-3 | This study constructed |
| pC9sgR-yA-G601-4 | This study constructed |
| pTR-C9sgR-yA-G500-2 | This study constructed |
| pTR-C9sgR-yA-G500-3 | This study constructed |
| pTR-C9sgR-yA-G500-4 | This study constructed |
| pTR-C9sgR-yA-G601-2 | This study constructed |
| pTR-C9sgR-yA-G601-3 | This study constructed |
| pTR-C9sgR-yA-G601-4 | This study constructed |

Table S3 The mutations involved in high-yielding strains. fs means frameshift, * means Stop gained, del means deletion, Ter means stop codon, and ext* means stop lost. The selected genes for validation are marked in orange shading.

| **No.** | **Genes** | **Type** | **Mutations** |
| --- | --- | --- | --- |
| 1 | AO090009000606 | SNP | Ala579Thr |
| 2 | AO090005001154 | SNP | Tyr44Ser |
| 3 | AO090005000961 | SNP | Leu1422Pro |
| 4 | AO090005000675 | SNP | Val471Ala |
| 5 | AO090001000199 | SNP | Pro178Leu |
| 6 | AO090001000237 | INDEL | Pro11fs |
| 7 | AO090001000261 | INDEL | Val209fs |
| 8 | AO090001000364 | SNP | Glu165Lys |
| 9 | AO090001000601 | INDEL | Glu337del |
| 10 | AO090003000100 | SNP | Ala256Thr |
| 11 | AO090003000209 | INDEL | Lys192fs |
| 12 | AO090003000220 | INDEL | Arg90fs |
| 13 | AO090003000635 | INDEL | Glu124fs |
| 14 | AO090003000984 | SNP | Ter280Lys and ext* |
| 15 | AO090023000107 | SNP | Tyr52Cys |
| 16 | AO090023000247 | SNP | Asp119Asn Cys113Tyr Asn111Asp |
| 17 | AO090023000249 | SNP | His41His Gln32* Leu29Ser Ser20Leu Gly13Asp Gly13Ser Glu12Lys Gln11* Pro2Ser Met1lys |
| 18 | AO090023000549 | SNP | Asp308Ala |
| 19 | AO090026000500 | SNP | Val239Ile |
| 20 | AO090026000066 | SNP | Phe330Ser |
| 21 | AO090026000034 | SNP | Ser431Leu |
| 22 | AO090012000933 | SNP | Asn49Asp |
| 23 | AO090166000031 | SNP | Glu23* |
| 24 | AO090113000116 | SNP | Met1lys |
| 25 | AO090020000240 | SNP | Lys432Glu |
| 26 | AO090038000001 | SNP | Ile323Ile Tyr324Asp Ter327Ter |
| 27 | AO090011000163 | INDEL | Lys32fs |
| 28 | AO090011000694 | SNP | Arg26His |
| 29 | AO090010000247 | INDEL | Glu499del |

Table S4 The result of Gene Ontology (GO) enrichment.

| **Ontology** | **Description** | **Id** | **Significant** | **Annotated** | **P value** | **Q value** |
| --- | --- | --- | --- | --- | --- | --- |
| biological process | multicellular organismal process | GO:0032501 | 1(7) | 43(3719) | 0.0782 | 1 |
| biological process | developmental process | GO:0032502 | 1(7) | 313(3719) | 0.4599 | 1 |
| biological process | multi-organism process | GO:0051704 | 1(7) | 323(3719) | 0.4709 | 1 |
| biological process | cellular process | GO:0009987 | 7(7) | 3373(3719) | 0.5045 | 1 |
| biological process | reproductive process | GO:0022414 | 1(7) | 383(3719) | 0.533 | 1 |
| biological process | metabolic process | GO:0008152 | 6(7) | 2951(3719) | 0.5588 | 1 |
| biological process | reproduction | GO:0000003 | 1(7) | 415(3719) | 0.5635 | 1 |
| biological process | negative regulation of biological process | GO:0048519 | 1(7) | 612(3719) | 0.7163 | 1 |
| biological process | positive regulation of biological process | GO:0048518 | 1(7) | 640(3719) | 0.7337 | 1 |
| biological process | biological regulation | GO:0065007 | 2(7) | 1537(3719) | 0.8583 | 1 |
| biological process | establishment of localization | GO:0051234 | 1(7) | 959(3719) | 0.8763 | 1 |
| biological process | response to stimulus | GO:0050896 | 1(7) | 1085(3719) | 0.9108 | 1 |
| biological process | localization | GO:0051179 | 1(7) | 1134(3719) | 0.9218 | 1 |
| biological process | regulation of biological process | GO:0050789 | 1(7) | 1288(3719) | 0.9492 | 1 |
| biological process | cellular component organization or biogenesis | GO:0071840 | 1(7) | 1622(3719) | 0.982 | 1 |
| cellular component | organelle | GO:0043226 | 6(6) | 2974(3545) | 0.3483 | 1 |
| cellular component | organelle part | GO:0044422 | 4(6) | 2028(3545) | 0.4865 | 1 |
| cellular component | protein-containing complex | GO:0032991 | 3(6) | 1612(3545) | 0.5681 | 1 |
| cellular component | membrane part | GO:0044425 | 1(6) | 861(3545) | 0.8119 | 1 |
| cellular component | membrane-enclosed lumen | GO:0031974 | 1(6) | 930(3545) | 0.8391 | 1 |
| cellular component | cell | GO:0005623 | 6(6) | 3467(3545) | 0.875 | 1 |
| cellular component | cell part | GO:0044464 | 6(6) | 3467(3545) | 0.875 | 1 |
| cellular component | membrane | GO:0016020 | 1(6) | 1183(3545) | 0.9127 | 1 |
| molecular function | enzyme regulator activity | GO:0030234 | 1(5) | 178(3227) | 0.2471 | 1 |
| molecular function | structural molecule activity | GO:0005198 | 1(5) | 187(3227) | 0.2582 | 1 |
| molecular function | molecular function regulator | GO:0098772 | 1(5) | 209(3227) | 0.2847 | 1 |
| molecular function | catalytic activity | GO:0003824 | 3(5) | 1913(3227) | 0.6702 | 1 |
| molecular function | binding | GO:0005488 | 1(5) | 1618(3227) | 0.9693 | 1 |

Table S5 The result of euKaryotic Ortholog Groups (KOG) enrichment.

| **Description** | **Significant** | **Annotated** | **P value** | **Q value** | **Symbol** |
| --- | --- | --- | --- | --- | --- |
| General function prediction only | 4(13) | 1087(5626) | 0.231920672 | 0.573644775 | AO090038000001  AO090001000261  AO090005001154  AO090012000933 |
| Carbohydrate transport and metabolism | 2(13) | 414(5626) | 0.247431159 | 0.573644775 | AO090005000675  AO090026000034 |
| Lipid transport and metabolism | 2(13) | 454(5626) | 0.2828354 | 0.573644775 | AO090023000549  AO090005000961 |
| Secondary metabolites biosynthesis, transport and catabolism | 2(13) | 495(5626) | 0.31923819 | 0.573644775 | AO090001000261  AO090020000240 |
| Replication, recombination and repair | 1(13) | 181(5626) | 0.346605345 | 0.573644775 | AO090001000601 |
| Transcription | 1(13) | 252(5626) | 0.449204516 | 0.573644775 | AO090001000601 |
| Function unknown | 1(13) | 266(5626) | 0.467589589 | 0.573644775 | AO090003000100 |
| Translation, ribosomal structure and biogenesis | 1(13) | 300(5626) | 0.509906466 | 0.573644775 | AO090001000364 |
| Signal transduction mechanisms | 1(13) | 407(5626) | 0.623670625 | 0.623670625 | AO090026000500 |
